# Microbial biocatalysis databases: An annotated selection of World Wide Web sites relevant to the topics in *microbial biotechnology*

**DOI:** 10.1111/1751-7915.13257

**Published:** 2018-02-14

**Authors:** Lawrence P. Wackett

**Affiliations:** ^1^ Department of Biochemistry, Molecular Biology & Biophysics BioTechnology Institute University of Minnesota St. Paul MN 55108 USA

## Biocatalysis/Biodegradation database

http://eawag-bbd.ethz.ch

The Biocatalysis/Biodegradation database (BBD) has compiled information on microbial enzyme‐catalyzed reactions that are useful for synthetic purposes or function in biodegradation pathways to remove chemicals from environmental compartments.

## Pathway Prediction System

http://eawag-bbd.ethz.ch/predict/

The Pathway Prediction System (PPS) is linked to from the BBD and uses metabolic rules to predict plausible metabolic pathways by which chemical compounds might be biodegraded by microorganisms.

## RAPID

http://rapid.umn.edu/rapid/

The RAPID database and tool provides information on enzyme catalysis, and there is developing tool provided that is focused on identifying substrate‐enzyme compatibilities.

## BRENDA

https://www.brenda-enzymes.org

BRENDA is an enzyme information system. It covers sources, substrates, inhibitors, kinetics and other characteristic features of the enzymes.

## BioCyc

https://biocyc.org

Bio/Cyc is an umbrella for different databases focused on metabolic pathways and genomes. It can be used to look at specific biocatalysis reactions or can be focused on the metabolism of specific microorganisms.

## Biocatalysis chemistry database: Accelrys

http://accelrys.com/products/datasheets/biocatalysis.pdf

This is a commercial database that is focused on the use of enzymes and microorganisms for chemical synthesis, which might offer advantages of selectivity, efficiency and greater environmental friendliness.

## EnviPath

https://envipath.org

EnviPath is a database that focuses on microbial degradation of organic chemicals in the environment.

## EMBL biocatalysis links

http://identifiers.org/registry?query=enzyme

This page created by the European Bioinformatics Institute contains numerous links to sites and databases relevant to biocatalysis.

## KEGG Ligand

http://www.genome.jp/kegg/kegg.html

The KEGG databases cover many aspects of cell function. It has extensive coverage of biochemical reactions, genes and enzyme nomenclature.

## Catalytic site atlas

http://www.ebi.ac.uk/thornton-srv/databases/CSA/

The catalytic site atlas compiles information on enzyme active sites and catalytic residues as derived from the X‐ray structures of enzymes.

## ExplorEnz database

http://www.enzyme-database.org

This enzyme database is organized along the lines of the Enzyme Commission classification system of enzyme reaction types.

## Peroxibase

http://peroxibase.toulouse.inra.fr

Peroxidases are useful enzymes and this website focuses on peroxidase enzymes, both their sequences and their reactions.

## CAZy:Carbohydrate‐active enzymes database

http://www.cazy.org

There are many commercially‐relevant reactions with carbohydrates and this database is dedicated to information on carbohydrate‐active enzymes.

## Biocatalytic synthesis links

http://biocatalysis.uni-graz.at/sites/links.html

This page contains numerous links to sites on chemistry and biochemistry that is relevant to biocatalysis.

## BioCatNet

https://biocatnet.de

The BioCatNet is a site that contains links to a collection of family‐specific enzyme databases, with a focus on enzymes of interest for biocatalysis.

## PDB

https://www.rcsb.org

The Protein DataBank (PDB) is a major resource for information on protein structures, typically determined by X‐ray crystallography or NMR methods.

## PDBsum

http://www.ebi.ac.uk/thornton-srv/databases/cgi-bin/pdbsum/GetPage.pl?pdbcode=index.html

PDBsum is a pictorial database providing a visual overview of material from the PDB, showing protein, ligands, metals, etc.

## Lipase database

http://www.au-kbc.org/beta/bioproj2/

Lipases have proven to be very versatile on the synthesis and chiral resolution of molecules, particularly in the pharmaceutical industry. This database focuses on information pertaining to lipases.

## Pfam database

http://pfam.xfam.org

The Pfam database focuses on protein sequences within large protein families. It represents the data using multiple sequence alignments and hidden Markov models.

## Cytochrome P450 engineering database

https://cyped.biocatnet.de

Cytochrome P450 enzymes are highly versatile because of their broad specificity and engineerability. This site focuses on a broad range of cytochrome P450 enzymes.

